# Large-Scale Genome Scanning within Exonic Regions Revealed the Contributions of Selective Sweep Prone Genes to Host Divergence and Adaptation in *Magnaporthe oryzae* Species Complex

**DOI:** 10.3390/microorganisms9030562

**Published:** 2021-03-09

**Authors:** Guohua Duan, Jiandong Bao, Xiaomin Chen, Jiahui Xie, Yuchan Liu, Huiquan Chen, Huakun Zheng, Wei Tang, Zonghua Wang

**Affiliations:** 1 State Key Laboratory for Ecological Pest Control of Fujian and Taiwan Crops, The School of Life Sciences, Fujian Agriculture and Forestry University, Fuzhou 350002, China; 2170204001@fafu.edu.cn (G.D.); baojd@fafu.edu.cn (J.B.); xiaominchen@fafu.edu.cn (X.C.); bbxjh1994@163.com (J.X.); liuyuchan1993@163.com (Y.L.); chenhuiquan2569@163.com (H.C.); huakunzheng@163.com (H.Z.); 2 Fujian Universities Key Laboratory for Plant-Microbe Interaction, College of Plant Protection, Fujian Agriculture and Forestry University, Fuzhou 350002, China; 3 Fuzhou Institute of Oceanography, Minjiang University, Fuzhou 350108, China

**Keywords:** *Magnaporthe oryzae*, population differentiation, host adaptation, selective sweep

## Abstract

*Magnaporthe oryzae*, one of the most notorious plant pathogens in the agronomic ecosystem, causes a destructive rice blast disease around the world. The blast fungus infects wide arrays of cultivated and non-cultivated plants within the Poaceae. Studies have shown that host speciation exerts selection pressure that drives the evolution and divergence of the *M. oryzae* population. Population genetic relationship deducted by genome-wide single nucleotide polymorphisms showed that *M. oryzae* differentiation is highly consistent with the host speciation process. In particular, the rice-infecting population of *M. oryzae* is distinct from populations from other hosts. However, how genome regions prone to host-mediated selection pressures associated with speciation in *M. oryzae*, especially at a large-scale population level, has not been extensively characterized. Here, we detected strong evidence of sweep selection throughout the genomes of rice and non-rice pathotypes of *M. oryzae* population using integrated haplotype score (iHS), cross population extended haplotype homozygosity (XPEHH), and cross population composite likelihood ratio (XPCLR) tests. Functional annotation analyses of the genes associated with host-mediated selection pressure showed that 14 pathogenicity-related genes are under positive selection pressure. Additionally, we showed that 17 candidate effector proteins are under positive and divergent selection among the blast fungus population through sweep selection analysis. Specifically, we find that a divergent selective gene, *MGG_13871*, is experiencing host-directed mutation in two amino acid residues in rice and non-rice infecting populations. These results provide a crucial insight into the impact of selective sweeping on the differentiation of *M. oryzae* populations and the dynamic influences of genomic regions in promoting host adaptation and speciation among *M. oryzae* species.

## 1. Introduction

Rice is cultivated globally and feeds more than a half of the world’s people [1]. *Magnaporthe oryzae*, the causal agent of rice blast disease, is a pathogenic filamentous fungus that causes a 10–30% loss of harvest each year [2,3]. *M. oryzae* is a multihost pathogen that can infect approximately 50 species of both wild and cultivated plants, especially cereals from the Poaceae family, such as rice, wheat, foxtail millet, and finger millet crops [4], posing a huge threat to global food security [5,6]. Since the whole genomic sequence of *M. oryzae* laboratory strain 70–15 was first deciphered in 2005 [7], many more isolates have been sequenced from different host plants, providing extensive resources for further research. Although the *M. oryzae* complex consists of genetically distinct lineages, divergence tends to be associated with host specialization [8]. In particular, the rice-infesting isolates come from a single lineage distinct from the others.

The limitation of gene flow among the different host populations is also partly responsible for *M. oryzae’s* host-specific subgroup divergence. For instance, it is clear that the *Oryza sativa* population of *M. oryzae* is different from others due to the lack of gene flow under asexual reproduction [9]. Nerveless, ancient recombination events and contemporary sexual reproduction might possibly occur within the *Oryza* isolate population [10,11]. As a seed-borne pathogen, *M. oryzae* can cause novel infections following seed and grain transportation both between and within countries. For instance, after wheat blast disease caused by *M. oryzae* isolates was first discovered in southern Brazil [12], neighboring countries such as Argentina, Bolivia, and Paraguay also detected it successively. Gene flow between different hosts has also been proposed to occur between divergent cereal- and grass-specific lineages of *M.oryzae* [8].

The rice blast pathogen *M. oryzae* engages in plant–pathogen interactions and a form of host shift. Host jumping between distant hosts, host range expansion, and host tracking between wild and domesticated hosts are sophisticated mechanisms that drive the evolutionary trend in plant pathogenic microbes [13,14,15,16]. Therefore, differentiation occurs among populations of *M. oryzae* from a variety of hosts that contribute to the formation of host-specific subgroups, including *Oryza* spp., *Setaria* spp., and *Triticum* spp. [17,18]. For instance, reports have shown that *M. oryzae* isolates from foxtail millet could also infect wild *Setaria* spp., indicating that these wild grass species serve as an alternative host that shelters the blast fungus during off seasons [19]. However, the inability of cultivated rice-infecting isolates from *M. oryzae* populations to infect non-cultivated wild grasses (ancestral rice species) gives credence to the postulations that *M. oryzae* experienced a host jump along with rice domestication approximately 10,000 years ago [4]. Domestication pressure appears to have narrowed the host range of rice-infecting *M. oryzae* isolates [11], which makes them distant from the *Setaria*-infecting isolates regarded as the closest relative of rice-infecting *M. oryzae* isolates [17]. Additionally, gene gain and loss resulted from the reshuffling of transposable elements throughout the individual genomes have been proposed as crucial contributors to host divergence between *Magnaporthe* species adapted to cultivated rice and *Setaria* [20]. Meanwhile, evolutionary relationship studies of *M. oryzae* isolates from multiple hosts have confirmed that speciation among *Magnaporthe* spp. coincides with the divergence of time-differing hosts [21]. To understand the evolutionary mechanisms that support the adaptation of *M. oryzae* to different hosts, it is crucially important to provide further insights into the sustainable management of rice blast disease. Although the mechanism underlying *M. oryzae* harbored by diverse hosts is still unknown, research on genomic regions that accelerate molecular evolution [22] and gene expression differentiation [9] provides an enlightening way to interpret the pathogen’s host adaptation.

Effector proteins secreted by the plant pathogen determine its pathogenicity or virulence. Therefore, there is an inevitable relationship between effector repertoire and pathogen–host specialization. It has been concluded that the specific expression patterns of effectors in three rice isolate lineages may be associated with the adaptation of *M. oryzae* to rice [23]. Similarly, during the interaction between barley and various host-specific lineage isolates, the effector *PWT1* acts as the major host-specific gene involved in infection [24], just as the *PWL1* and *PWL2* genes are involved in *M. oryzae’s* infection of weeping lovegrass [25]. However, the secreted effector *AVR1-CO39* cloned from grass isolate 2539 can be avirulence in rice with no such functional genes [26,27,28]. Directional selection induced by the various hosts also acts as a major evolutionary force that results in the host specificity of *M.oryzae* [29]. Comparative genomic analysis of *M. oryzae* and *M. penniseti* has suggested that the divergence of pathogenicity-related gene repertoires contributes to host adaptation [30].

Selective sweep is regarded as an essential genetic mechanism for enabling species to successfully adapt to prevailing environmental conditions by fixing novel mutations associated with beneficial phenotypic traits. This provides straightforward evidence of the evolution of populations. Diverse statistical theories and tools have been developed to identify genome-wide selection sweep events, such as F-statistics (Fst) [31], Tajima’s D-test [32], the cross-population composite likelihood ratio (XPCLR) [33], the integrated haplotype score (iHS) [34], and cross-population extended haplotype homozygosity test (XPEHH) [35]. iHS was used in a single population (within-population method) to reveal selection evidence according to the theory of linkage disequilibrium, which is effective for regions containing sites with a rapidly increased frequency of the derived allele [36]. The XPEHH and XPCLR are widely used to detect the selection footprint between two populations. Based on the difference in the multi-locus allele frequency of two populations, XPCLR shows sensitive performance regarding allele frequency fluctuations at loci with random drift [33]; in contrast, XPEHH uses linkage disequilibrium to distinguish the selection region [35]. The XPCLR, iHS, and XPEHH are widely employed to uncover genome-wide selective sweep regions. For instance, a host-driven selective sweep was identified by the XPEHH, XPCLR, and iHS tests in populations of the fungal wheat pathogen *Zymoseptoria tritici* from two different wheat cultivars [37]. Selective sweep throughout the genome, as detected by iHS and the XPEHH, was considered a more potent contributing force to barley scald pathogen *Rhynchosporium commune* through local adaptation [38]. It is likely that for the rice pathogen Xanthomonas oryzae pv. oryzae, selective sweep also had a substantial impact on the formation of the population structure [39]. The iHS, XPEHH, and XPCLR were adopted to detect the selection footprint on the X chromosome in three geographical pig breeds [40], and positive selection genes involving climate adaptation were previously identified in African cattle populations with the XPEHH and XPCLR tests [41]. The iHS, XPEHH, and XPCLR tests also revealed that positive or divergent selection genes are associated with adaptive traits in weedy rice [42]. Although genetic and environmental homogeneity in agricultural ecosystems imposes intense and uniform selection pressures on plant pathogens, the impact on the genomic variation recorded among *M. oryzae* populations is not fully understood.

To investigate the evolutionary mechanism that caused host adaption to emerge for *M. oryzae*, we collected 197 whole-genome sequences of isolates from ten different hosts, including rice, wheat, and cultivated and non-cultivated grasses. Above all, genome-wide evolution relationship analysis of *M. oryzae* populations revealed that host specialization crucially impacts the population structure. We showed that *M. oryzae* populations from rice are distinct from isolates from non-rice hosts. Furthermore, a composite strategy including the iHS, XPEHH, and XPCLR tests was adopted to identify the selective sweep within and between rice and non-rice *M. oryzae* populations. The selected region, spanning genes with strong positive selection signals, includes pathogenicity-related genes, such as glycosyl hydrolase, glucanase, and cutinase. Meanwhile, population-specific selective effector candidates, including *MGG_15370, MGG_07993, MGG_00230, MGG_07352, MGG_06231, MGG_06234, MGG_17666, MGG_15458, MGG_05538, MGG_14374, MGG_08214, MGG_16925, MGG_15106, MGG_16938, MGG_07311, MGG_07246,* and *MGG_16953*, were also identified in rice and non-rice populations. This study expands our understanding of host-driven population differentiation in plant–pathogen interactions.

## 2. Materials and Methods

### 2.1. Identification of Single Nucleotide Polymorphisms (SNPs)

Nucleotide sequences of 197 *M. oryzae* isolates previously sequenced from 10 different hosts (Supplementary Table S1) were downloaded from the National Centre for Biotechnology Information (NCBI). Subsequent comparative genomic analyses with the Mummer4 software (–maxmatch –c 100 –p) [43] identified SNPs between the 70-15 strain and the individual isolates. The SnpEff [44] tool was used to precisely extract SNPs located in the exonic regions of the 70-15 strain. VCFtools was applied to filter the variants with the following main options: –max-missing 0.50 –mac 3 –max-alleles 2 [45].

### 2.2. Phylogeny and Population Structure of the M. oryzae Species Complex

A neighbor-joining (NJ) tree was constructed using a Provesti’s distance [46] of 1000 bootstrap replication with the poppr package in R-3.6.1 [47]. A no-root tree with bootstrap values was visualized with the ggtree package [48]. Next, the relationships between different host-derived isolate populations were analyzed with principal component analysis (PCA) in the adegenet package [49]. The population structure was analyzed with the discriminant analysis of the principal component method using the adegenet package in R-3.6.1 [49].

### 2.3. Population Selective Sweep Detection

The iHS [36] and XPEHH [50] were calculated to detect the selective signature for rice and other host populations in the rehh packages in R [51]. For a better comparison of selection signals, |iHS| scores were transformed into −log_10_(2Φ(-|iHS|), in which Φ (*x*) is the cumulative Gaussian distribution function. Selection pressure on rice- and non-rice-infecting *M. oryzae* isolates at the individual population level was analyzed with the iHS test [52]. A resulting iHS greater than 0 with a *p*-value of ≤0.01 indicated positive selection. The XPEHH test was used to examine the comparative selection pressure between populations of rice- and non-rice-infecting isolates. The XPCLR [33] was used to detect and compare potential selective regions between rice- and non-rice-infecting populations. A resulting XPEHH value greater than 0 indicated rice-infecting *M. oryzae* population with selection pressure. On the contrary, values lower than 0 implied a selection in the non-rice infecting population. For the iHS and XPEHH tests, *p*-values were computed using the −log_10_ scale; thus, values greater than 2 were considered candidate selection sites. The top 1000 normalized XPCLR values for each window region in the XPCLR test represented a selection region.

### 2.4. Candidate Genes and Functional Annotation

Each SNP and sliding window region with genomic position and selective scores were calculated by the iHS, XPEHH, and XPCLR tests. According to the annotated reference genome file from the NCBI database, the potentially selective SNPs were remapped to the reference genomic genes. Amino acid sequences of candidate genes with significant selective signal SNPs were submitted to the Pathogen Host Interaction (PHI) database [53], the non-redundant (Nr) protein database (http://www.ncbi.nlm.nih.gov/ (accessed on 1 January 2021)), Gene Ontology (GO) (http://www.geneontology.org (accessed on 1 January 2021)), the Universal Protein Resourcce (UniProt) (https://www.uniprot.org (accessed on 1 January 2021)), Protein family (Pfam) database (http://pfam.xfam.org/ (accessed on 1 January 2021)), and eggnog database (http://eggnogdb.embl.de/ (accessed on 1 January 2021)) for functional prediction.

### 2.5. Effector Candidate Prediction

Gene amino acid sequences under selection were also submitted to SignalP-5.0 [54], TargetP 2.0 [55], and EffectorP [56] to predict potential effectors. SignalP-5.0 (http://www.cbs.dtu.dk/services/SignalP/ (accessed on 1 January 2021)) was adopted to predict the presence and location of signal peptide cleavage sites. The subcellular localizations of putative candidate effector proteins identified in this study were predicted with TargetP 2.0 (http://www.cbs.dtu.dk/services/TargetP/ (accessed on 1 January 2021)). EffectorP 2.0 was trained to detect the effector candidates in fungi. As the threshold to filter the obscure genes predicted, a 0.6 probability was used.

### 2.6. Host Directional Mutation

The amino acid sequence of selection associated gene *MGG_13871* was extracted from each *M. oryzae* genome. Multiple sequence alignment was implemented in the ClusterW program in MEGA X [57]. Multiple sequence alignment of *MGG_13871* amino acid sequences from rice- and non-rice-infecting populations was visualized by the ggmsa package in R (https://github.com/YuLab-SMU/ggmsa (accessed on 1 January 2021)).

## 3. Results

### 3.1. Population Genomic Divergence Driven by Host Adaptation in the M. oryzae Species Complex

**Figure 1:**
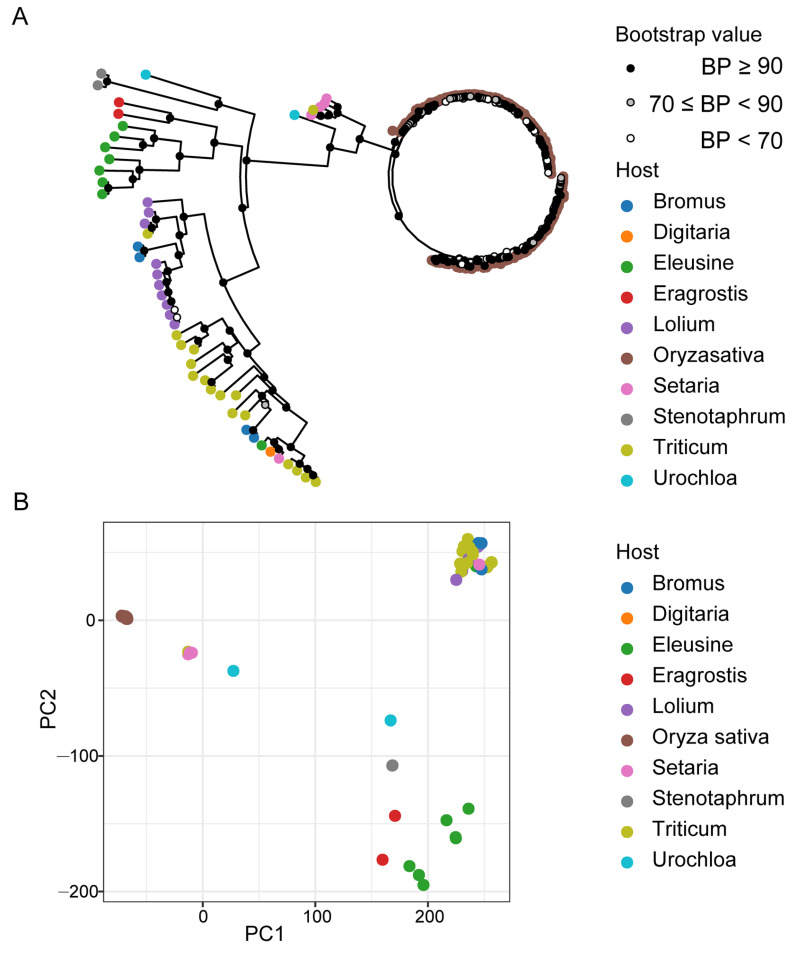
Phylogenetic relationship of M. oryzae isolates from different host plants. (**A**) Neighbor-joining phylogenetic tree based on the whole-genome exon region single-nucleotide polymorphism (SNP) data from 197 isolates among 10 different hosts. The dot in white, grey, and black (nodes) indicates bootstrap support greater than 90%, between 70% and 90%, and lower than 70% after 1000 replications, respectively. (**B**) Two dimensions principal component analysis (PCA) showing the relationship of different hosts infesting *M. oryzae* populations. Colorful dots at the tips of branches mark the isolates from a variety of hosts.

To evaluate the dynamics in the genomic structures of the 197 *M. oryzae* isolates collected from 10 different hosts, we assessed the impacts of divergent genomic features on the adaptation of *M. oryzae* isolates to different hosts. We identified a total of 366,502 bi-allelic SNP loci in the exon regions within genomes. Phylogenetic analysis, principal component analysis (PCA), and population structure component analysis unraveled the relationship between different host-infecting *M. oryzae* populations. These analyses revealed significant differences between populations of *M. oryzae* isolates from different host plants. The evolutionary trend analyses revealed that the 197 *M. oryzae* isolates obtained from the 10 different hosts can be clustered in two major clades, with the first clade (clade-1) comprising rice-infecting isolates and the second clade (clade-2) containing non-rice-infecting isolates (Figure 1A). Moreover, higher bootstrap values were obtained for isolates from common host plants than isolates from distant host plants.

We also observed a lesser genetic distance within clades consisting of rice-infecting isolate clades compared to clades containing isolates capable of infecting multiple hosts. The topological pattern obtained from the phylogenetic analysis further showed the aggregation of selected isolates from rice-infecting population with lesser evolutionary separation into distinct clusters, even under principal components 1 and 2 (PC1 and PC2) resolutions. The isolates adapted to non-rice hosts showed distant and dispersed topological distribution patterns (Figure 1B).

**Figure 2:**
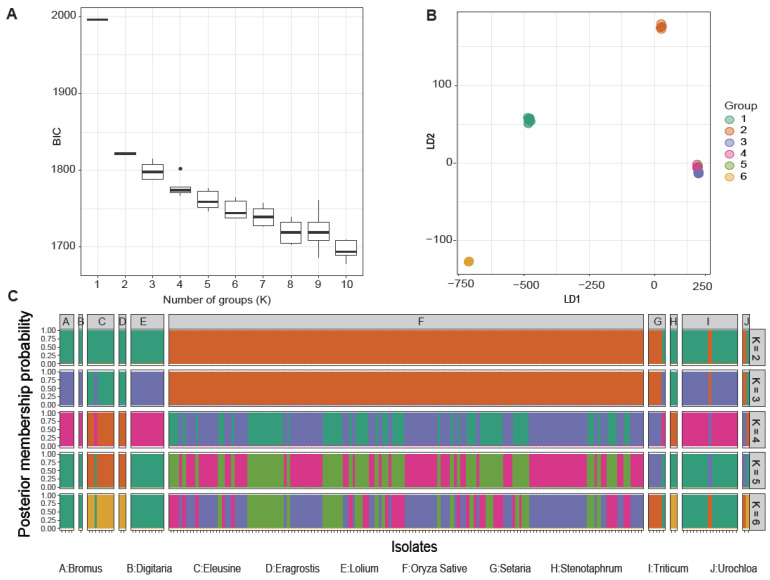
Analysis of the population genetics of *Magnaporthe oryzae* according to the discriminant analysis of principal components (DAPC). (**A**) The Bayesian information criterion (BIC) distribution. (**B**) Scatter plot of the first (LD1) and second (LD2) linear discriminant functions with group numbers from 2 to 6. Dots with different colors represent each sample that was assigned to a designated group. (**C**) Bar plots of the posterior membership probabilities acquired from the DAPC analysis with K from 2 to 6. Each isolate is represented by one bar.

We examined the optimal number of clusters with the discriminant analysis of principal components (DAPC) based on the lowest associated Bayesian information criterion [58] and linear discriminants analysis. These analyses showed that the best number of clusters unambiguously correlated with the host preference of individual isolates within distinct populations (Figure 2A,B). However, cluster number adjustments resulted in the formation of marginal groups, with rice-infecting populations and non-rice-infecting populations having two groups each. The first group of the non-rice-infecting population predominantly consisted of isolates from *Bromus* spp., *Digitaria* spp., *Lolium* spp., and *Triticum* spp., while the other non-rice-infecting population comprised isolates that infect *Eleusine* spp., *Eragrostis* spp., and *Stenotaphrum secundatum* (Figure 2C). Regardless, an admixture of genetic signatures was present among the distinct *M. oryzae* populations.

### 3.2. Selective Sweep Signatures in the Genomic Sequences of M. oryzae Populations

**Figure 3:**
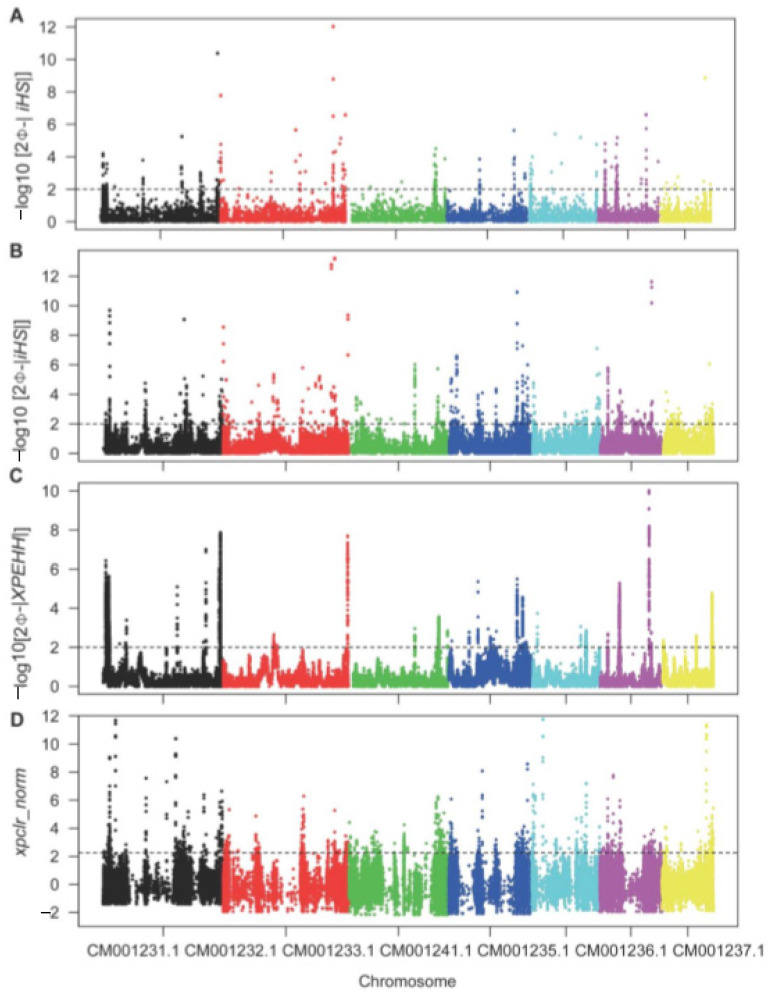
Scatter plot of genome-wide selective signals in the integrated haplotype score (iHS) and the cross-population extended haplotype homozygosity (XPEHH) and cross-population composite likelihood ratio (XPCLR) tests. (**A**) Genome-wide distribution of -log_10_ *p* of standardized iHS in rice isolate population. (**B**) Genome-wide distribution of −log_10_ *p* of standardized iHS in non-rice isolate population. (**C**) Genome-wide distribution of −log_10_ *p* of standardized XPEHH scores between rice and non-rice isolate populations. (**D**) Genome-wide distribution of normalized XPCLR scores with 500 bp sliding windows between rice and non-rice isolate populations.

To exhaustively mine the selective region of beneficial genes, we performed iHS, XPEHH, and XPCLR analyses to identify the candidate region by scanning the whole genome within and between populations. In terms of population structure, we assumed that natural selection acted differently on the rice- and non-rice-infecting *M. oryzae* populations in accordance with their host differences. Further examination of individual SNPs or window regions with selection signals from three independent tests revealed a nearly uniform distribution of the selective regions across each chromosome (Figure 3). Although most of the SNPs and window regions identified within the respective populations’ genomes were under neutral selection, outliers in each test were detected that were considered the selective sweep region. A single population-based iHS analysis revealed 263 and 412 genes with −log_10_ *p*-values of >2 in rice- and non-rice-infecting isolate populations, respectively, along with 1277 SNP sites that mapped to the exon regions of 97 genes (Supplementary Tables S2 and S3). Similarly, XPEHH analyses between rice- and non-rice-infecting populations identified 6632 SNPs located in the exon regions of 102 genes with −log_10_ *p*-values >2 (Supplementary Table S4). Furthermore, whole-genome sliding window analysis records obtained from XPCLR tests identified a total of 321 genes as the top 1000 normalized cross-population composite likelihood ratios between the two populations (Supplementary Table S5).

### 3.3. Selective Genes Associated with Host Speciation—Functional Annotation

We performed comparative functional annotation analyses of genes located within selective sweep regions on Nr, Pfam, Uniprot, eggnog, and PHI platforms to retrieve selection-associated genes. Although most of the genes recovered from selection prone regions of the genome have not been functionally characterized in rice blast fungus, our investigations revealed that 14 pathogenicity or virulence-associated genes, including the effector protein (Avr-Pita1) coding gene (*MGG_15370*) [59], had undergone positive selection in the non-rice-infecting population (XPEHH = −2.77) (Table 1). Positive selection of genes (*MGG_07528* and *MGG_07312*) is likely an essential genetic phenomenon that drives host-shift among non-rice-infecting populations (iHS = 2.83 and iHS = 3.19, respectively).

Furthermore, we observed that in the rice-infecting population, positive selection acted upon *MGG_04842* along with genes for pathogenicity and survival of the rice blast fungus (iHS = 2.92). Additionally, functional analyses showed that pathogenicity-related genes, including *MGG_14061*, *MGG_05464*, *MGG_10730*, *MGG_14767*, *MGG_02916,* and *MGG_17278*, were positively selected in both rice- and non-rice-infecting populations (Table 1). These investigations also showed that seven members of glycosyl hydrolase family encoded by *MGG_09738*, *MGG_09608*, *MGG_02911*, *MGG_00050*, *MGG_05489*, *MGG_16377*, and *MGG_07101*, along with mixed-linked glucanase (*MGG_07306*) [60,61], cell wall glycosyl hydrolase (*MGG_04305*) [62], and cutinase (*MGG_11091*) [63], are positively selected exclusively in non-rice-infecting population.

### 3.4. Candidate Effectors Experiencing Positive Selection

Effectors play a key role in host identification for *M. oryzae*, so it is essential to discover potential effectors harbored in the selective sweep region. Effector candidates were predicted within genes selected with SignalP 5.0, TargetP 2.0, and EffectorP2.0 based on amino acid sequences. Integrated prediction analysis identified 20 candidate effector proteins with probability scores above 0.6 (Table 2). Remarkably, only candidates *MGG_17666*, *MGG_15458*, *MGG_05538*, *MGG_00230*, *MGG_07352*, and *MGG_15370* showed positive selection status in non-rice-populations with iHS values of 2.63, 3.10, 3.91, 3.19, and 3.13 and an XPEHH score of −2.77, respectively. For rice-infecting population, candidates *MGG_08214*, *MGG_07993*, *MGG_06231*, *MGG_16925*, *MGG_15106*, *MGG_07246*, *MGG_16953*, and *MGG_14374* were under positive selection with an iHS of 5.15; XPCLR values of 2.26, 2.71, 2.70, 3.45, 2.73, and 2.31; and an XPEHH score of 3.08, respectively (Table 2). Meanwhile, *MGG_06234*, *MGG_16938*, and *MGG_07311* were selected for the rice- and non-rice-infecting populations. Additionally, diversified selection acted on the genes shared between rice- and non-rice-infecting populations. We also found that a hypothetical protein lacking the secretion signal motif selective gene (*MGG_13871*) experienced differential selection pressure in rice- and non-rice-infecting isolates. For instance, it was positively selected in the non-rice-infecting population (XPEHH = −3.14) but negatively selected in rice-infecting *M. oryzae* population (iHS = −2.97).

### 3.5. MGG_13871 Experienced Host Directional Mutation

**Figure 4:**
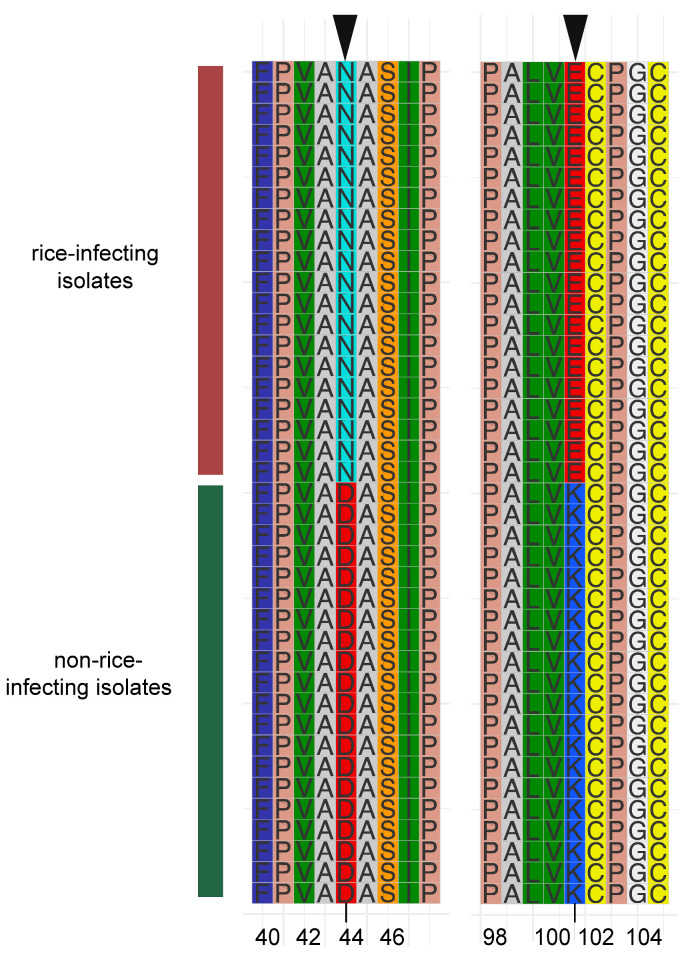
Multiple amino acid sequence alignment of the selective motif of a major positively selected gene (*MGG_13871*) in non-rice-infecting isolates. Motif sequences of the selectable regions of 20 isolates each from rice- and non-rice-infecting *M. oryzae* were extracted for comparative motif sequence analysis. The red and green vertical bars represent the rice and non-rice host isolates, respectively. The arrows on the top indicate the directed mutation residues. The number at the bottom indicates the mutation’s position in the gene.

Furthermore, to uncover the fixed mutations in genes associated with specific phenotypes that contributed to population differentiation under natural selection, we further extracted the amino acid sequence of *MGG_13871* from each genome of *M. oryzae* isolates in this study, because *MGG_13871* underwent divergent selection in the rice- and non-rice-infecting *M. oryzae* populations. Multiple alignments of *MGG_13871* amino acid sequences displayed host-directional mutations in two residues (Figure 4). At the 44th amino acid residue position, the amino acid was asparagine (N) in the rice-infecting population, but aspartic acid (D) in the non-rice-infecting population. Likewise, the lysine (K) in the rice-infecting population was substituted with lysine (K) in the non-rice-infecting population at the 102nd position. Taken together, we concluded that *MGG_13871* experienced host-directed mutation in rice- and non-rice-infecting populations.

## 4. Discussion

In this study, whole-genome SNPs were used to resolve the population structure and composition in *M. oryzae* populations derived from 10 various host plants, including rice, wheat, and grass. This study further confirmed previous research, in that reshuffling pathotypes from multiple lineages favored the formation of identical isolates with no common cryptic lineages [8]. Host specificity is a noticeable genetic characteristic that has been the focus of whole-genome sequencing and multi-locus analysis in previous studies to decipher the genomic parameters of the host diversity of isolates within the *M. oryzae* species complex [17,21].

This study demonstrated that rice-infecting *M. oryzae* isolates constitute a monophyletic clade with closer evolutionary distance than non-rice hosts. These results partly showed that rice-infecting isolates are under an almost uniform selection pressure from rice hosts due to limited genetic diversity or genomic reshuffling among rice cultivars. Furthermore, we asserted that rice-infecting isolates originated from a recent common ancestor through asexual reproduction. However, a small number of isolates within the non-rice-infecting population with an identical host form unique discrete clusters with a relatively wider phylogenetic distance. We deduced that more genetic diversity in the non-rice hosts exerts tremendous selection pressure or induces higher incidences of gene flow among non-rice-infecting isolates, especially in clusters containing mixtures of isolates from *Bromus* spp. and *Lolium* spp. These observations support previous research suggesting that genetic exchange is a crucial contributor to the minimization of divergence between lineage-specific *Magnaporthe* isolates from non-rice hosts [8].

Additionally, the population structure analysis conducted in this study showed that wheat-infecting isolates belong to the *M. oryzae* species complex. These findings further confirm previous positions that the divergence of wheat blast isolates does not qualify them as new species. Although rice-infecting isolates are phylogenetically distant from *Magnaporthe* isolates from other hosts, they share relatively closer evolutionary ties with isolates from *Setaria* [17]. Herein, we demonstrated the grouping of *Magnaporthe* isolates into two distinct groups: rice-infecting populations and non-rice-infecting populations [64].

Furthermore, the evaluation of gene flow patterns in rice-infecting *M. oryzae* populations within Asia identified mainland China as the primary source of gene flow. We also observed that genetic material of *M. oryzae* populations from mainland China and Japan possibly first converged in Taiwan, China, and later served as the gene pool for Korean isolates (Supplementary Figure S1). This may be attributed to the fact that mainland China is the origin of wild and domesticated rice [65]. We posited that rice-infecting *Magnaporthe* isolates possibly evolved along with their rice host in mainland China and were later introduced to subsequent regions, including Taiwan, China, and Korea. Although the gene flow was from mainland China to Taiwan, isolates from Taiwan display relatively greater evolutionary distance from isolates sampled from the neighboring Fujian province in China [64]. For the rice-infecting isolates, rice varieties also have a significant impact on *M. oryzae* population structure. Most of the isolates are from Japonica growing areas such as Taiwan, the north of China, and Japan, which are close to one another. In contrast, isolates sampled from *indica* growing areas, including the Fujian and Yunan provinces in China and India, frequently assembled into the same cluster (Supplementary Figure S2).

Host shift, host jump, and host expansion are adaptation characteristics that allow *M. oryzae* to successfully co-evolve to match agronomic changes (blast resistance) associated with the domestication of rice [4]. Previous studies have speculatively proposed that the blast fungus possibly undergoes accelerated genome reshuffling to support adaptive evolution, which enables it to experience host shift, host jump, or host expansion [22,24]. We identified, for the first time, credible selection-prone genomic regions containing protein-coding genes in rice- and non-rice-infecting populations through the integrated application of the iHS, XPEHH, and XPCLR tests. To eliminate bias resulting from high-frequency transposons in the genome of *Magnaporthe* spp., we only considered and compared mutations occurring in the exonic regions of individual isolates within the respective populations. Generally, pathogens retain survival or beneficial mutations during host adaptation induced speciation. Therefore, positively selected genes likely include genes that play crucial roles in the progression of host–pathogen interactions [66]. Effector proteins are small secreted proteins from fungal pathogens that have been shown to play dynamic roles in the progression of host–pathogen interactions by either facilitating host identification or acting as pathogenicity and/or virulence factors [67]. Additional studies have shown that candidate effector proteins in blast fungus undergo rapid evolution to yield new pathotypes of *M. oryzae* with broader host ranges or adaptations for specific hosts [68].

We showed that 17 candidate effector proteins undergo differential selection sweep in rice- and non-rice-infecting *M. oryzae* populations and speculatively concluded that the differential selection sweep of pathogenesis-related candidate effector proteins within and between rice- and non-rice-infecting *M. oryzae* populations likely accounts for host divergence and host specificity among *Magnaporthe* spp. We also showed that selection-prone genes, including *MGG_13871* and *MGG_07815*, experience differential host-specific mutations between rice- and non-rice-infecting populations. These two candidate selectable genes experienced uniform and synonymous amino acid substitution in rice- and non-rice-infecting populations. The site, amino acid, and type of selection pressure experienced by *MGG_13871* and *MGG_07815* varied between the two populations. For instance, *MGG_13871* experienced positive selection in non-rice-infecting populations and negative selection in rice-infecting populations. We accordingly concluded that *MGG_13871* likely constitutes a host specificity determiner in *Magnaporthe* spp. [69].

Meanwhile, specific selection prone candidate effectors in rice- and non-rice-infecting populations, including *MGG_17666*, *MGG_15458*, and *MGG_08435*, could also play additional roles in modulating population differentiation and host specificity between the distinct *Magnaporthe* populations reported in this study. Moreover, we also identified a cutinase gene, *MGG_11091*, in the selective sweep region, an essential enzyme required to degrade the host cuticle to support the successful invasion of blast fungus into host tissues. *MGG_05489* and *MGG_16377* experienced positive selection in rice-infecting populations. These two genes are members of the glycosyl hydrolase family that were previously implicated in the pathogenesis of *Magnaporthe* spp. However, when compared to rice-infecting isolates, wheat-infecting *M. oryzae* isolates seemingly favor the seed; therefore, positive selection of mixed-linked glucanase *MGG_07306* is possibly a host adaptation mechanism acquired by *M. oryzae.*

In summary, our results provide the crucial insight that selective sweeping plays a role in driving *M. oryzae* population differentiation associated with host adaptation, and we revealed potential genomic regions of interest to understand the genetic mechanisms of host specialization in *M. oryzae.*

## Figures and Tables

**Table 1:** Significantly selected pathogenicity-related genes identified in *M.oryzae* through iHS, XPEHH, and XPCLR analyses. PHI, Pathogen–Host Interaction database.

Gene	iHS (Non-Rice)		iHS (Rice)		XPEHH		XPCLR	PHI	Description	Identity (%)
	Score	−log *p*	Score	−log *p*	Score	−log *p*				
*MGG_15370*					−2.77	2.25		PHI:2150	effector	99.5
*MGG_07528*	2.83	2.34						PHI:121	Lost pathogenicity	100.0
*MGG_04842*			2.92	2.45				PHI:362 /PHI:2520	Lost pathogenicity /lethal	68.9
*MGG_07312*	3.19	2.85						PHI:2200 /PHI:3163	Lost pathogenicity /reduced_virulence	100.0
*MGG_02916*	3.03	2.61	2.81	2.31				PHI:4962	reduced_virulence	100.0
*MGG_02986*	2.69	2.15						PHI:893	reduced_virulence	100.0
*MGG_03087*	2.62	2.06						PHI:6409	reduced_virulence	91.3
*MGG_07514*	2.73	2.20						PHI:5440	reduced_virulence	100.0
*MGG_09396*	7.01	11.62						PHI:1097	reduced_virulence	67.5
*MGG_14061*			2.73	2.19				PHI:4509	reduced_virulence	66.1
*MGG_05464*							3.60	PHI:2208	reduced_virulence	100.0
*MGG_10730*							2.82	PHI:2095	reduced_virulence	100.0
*MGG_14767*							2.94	PHI:2049	reduced_virulence	100.0
*MGG_17278*							2.29	PHI:200	reduced_virulence	67.1

**Table 2:** Dynamic characteristics of putative effector proteins identified in selective regions of the genomes.

Gene	iHS (Non-Rice)		iHS (Rice)		XPEHH		xpclr_norm	SignalP	Subcellular Localization	EffectorP (%)
	Score	−log *p*	Score	−log *p*	Score	−log *p*				
*MGG_15370*					−2.77	2.25		+	extracellular	96.7
*MGG_07993*							2.26	+	extracellular	94.0
*MGG_00230*	3.19	2.84						+	extracellular	91.7
*MGG_07352*	3.13	2.75						+	extracellular	90.2
*MGG_06231*							2.71	+	extracellular	88.2
*MGG_06234*	2.97	2.53					2.25	+	extracellular	88.1
*MGG_17666*	2.63	2.06						+	extracellular	85.7
*MGG_15458*	3.10	2.71						+	extracellular	79.1
*MGG_05538*	3.91	4.03						+	extracellular	78.1
*MGG_14374*					3.08	2.68		+	extracellular	77.9
*MGG_08214*			5.15	6.57				+	extracellular	76.2
*MGG_16925*							2.70	+	extracellular	73.9
*MGG_15106*							3.45	+	extracellular	73.3
*MGG_16938*	3.80	3.85					6.23	+	extracellular	72.8
*MGG_07311*	3.17	2.82					2.84	+	extracellular	69.1
*MGG_07246*							2.73	+	extracellular	67.7
*MGG_16953*							2.31	+	extracellular	60.8

## Supplementary Materials

The following are available online at https://www.mdpi.com/2076-2607/9/3/562/s1, Figure S1: Gene flow evaluation in Asia; Figure S2: Phylogenetic tree of M. oryzae; Table S1: *M.oryzae* isolates’ host information; Table S2: Integrated haplotype score (iHS) test in rice host *M. oryzae* isolates; Table S3: iHS test in non-rice host *M. oryzae* isolates; Table S4: Cross-population extended haplotype homozygosity (XPEHH) test between rice and non-rice host *M. oryzae* isolates; Table S5: Cross-population composite likelihood ratio (XPCLR) test between rice and non-rice host *M.oryzae* isolates.
