# Supplementary material for: Large-Scale Genome Scanning within Exonic Regions Revealed the Contributions of Selective Sweep Prone Genes to Host Divergence and Adaptation in Magnaporthe oryzae Species Complex
Source: Microorganisms. 2021 Mar 9;9(3):562. doi: 10.3390/microorganisms9030562 (PMC8000120; doi:10.3390/microorganisms9030562)
Supplement: Supplementary file 1 [file microorganisms-09-00562-s001.zip › supplementary.pdf]

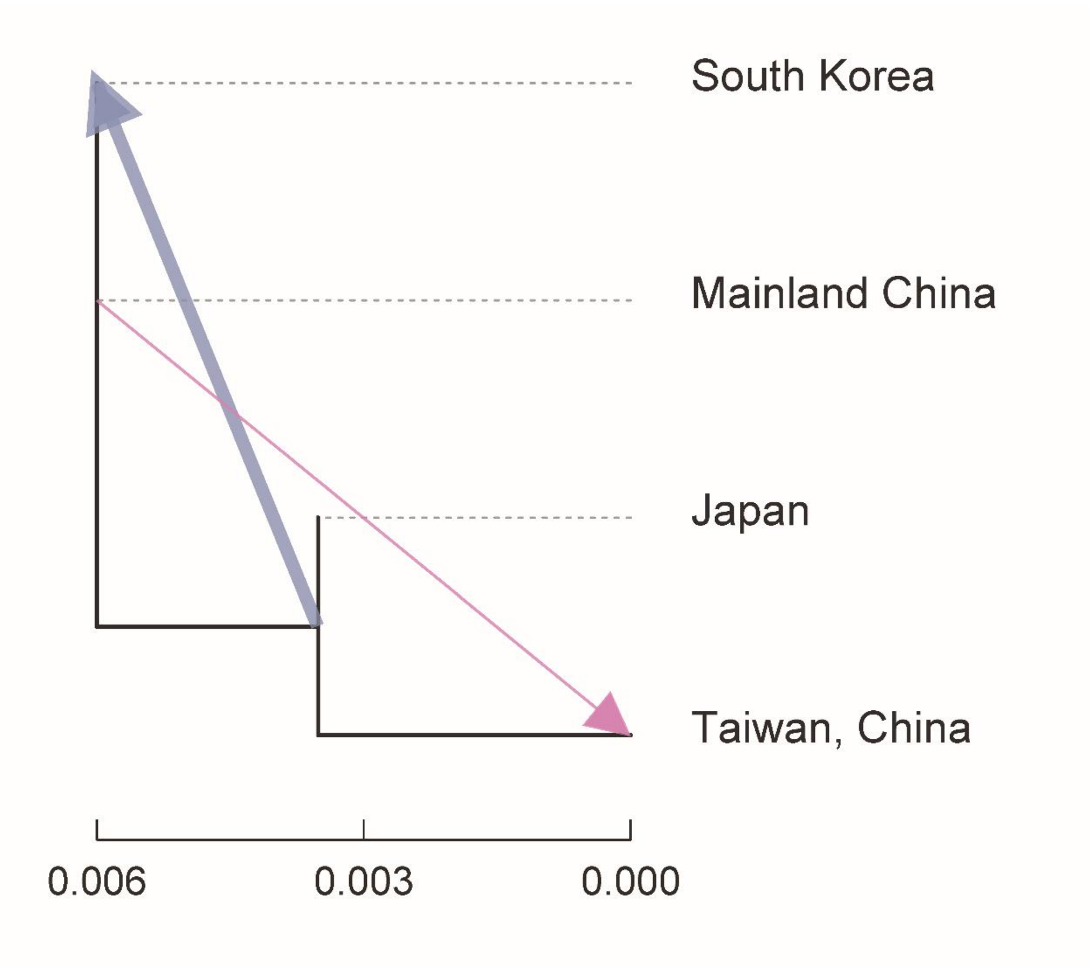

Figure S1 Gene flow evaluation in Asia at m=2 (two migration events). Arrow lines were colored presented migration events. Migration width represented the fraction of ancestry received from the donor population.

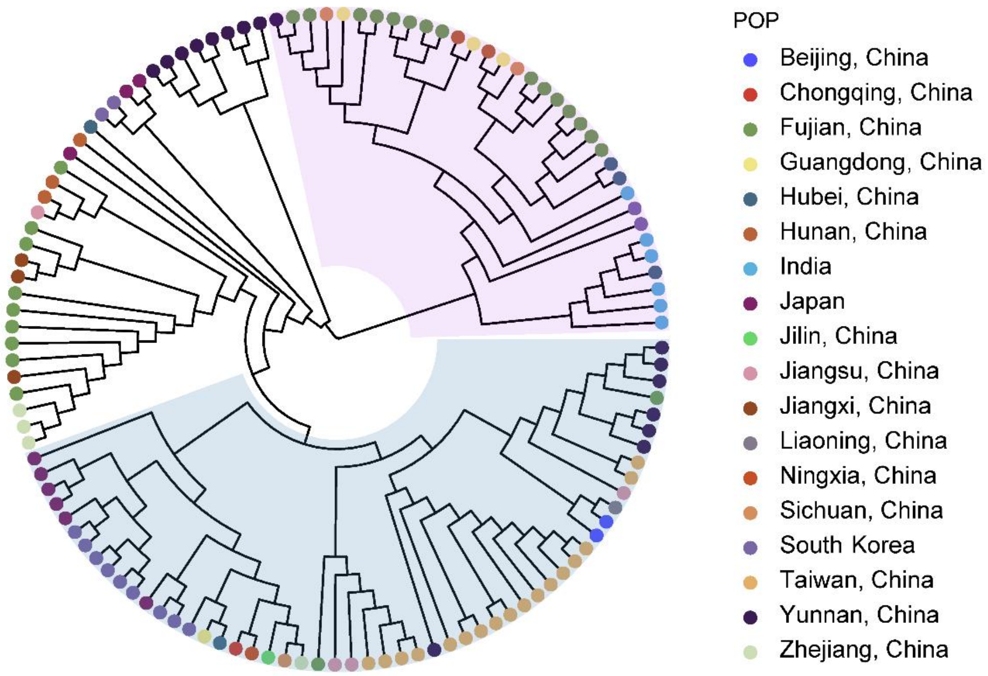

Figure S2 Neighbor-joining phylogenetic tree based on whole-genome coding region SNPs data of *M. oryzae* isolates from Asia. Dots in the tip of branches were colored according to sampling location. Branched under colored areas represented main two clades in the evolutionary tree.
